# Retraction: Arginine methylation of SHANK2 by PRMT7 promotes human breast cancer metastasis through activating endosomal FAK signalling

**DOI:** 10.7554/eLife.72188

**Published:** 2021-07-22

**Authors:** Yingqi Liu, Lingling Li, Xiaoqing Liu, Yibo Wang, Lingxia Liu, Lu Peng, Jiayuan Liu, Lian Zhang, Guannan Wang, Hongyuan Li, Dong-Xu Liu, Baiqu Huang, Jun Lu, Yu Zhang

Liu Y, Li L, Liu X, Wang Y, Liu L, Peng L, Liu J, Zhang L, Wang G, Li H, Liu D-X, Huang B, Lu J, Zhang Y. 2020. Arginine methylation of SHANK2 by PRMT7 promotes human breast cancer metastasis through activating endosomal FAK signalling. *eLife* **9**:e57617. doi: 10.7554/eLife.57617.

Published 26, April 2020

We are retracting the *eLife* paper cited above in which we suggested that PRMT7 promoted breast cancer cell metastasis through di-methylating SHANK2 R240. After publication, an expert in the field of PRMTs noted that we did not provide in vitro methylation/mapping data to demonstrate the direct methylation of SHANK2 by PRMT7. We re-performed experiments to detect the effect of PRMT5 on SHANK2 methylation, and the results indicate that PRMT5 might play an important role in SHANK2 di-methylation. Based on these results, we believe that the di-methylation of SHANK2 by PRMT7 might be dependent on PRMT5, which means our original conclusion that PRMT7 directly catalyses the SHANK2 di-methylation needs to be investigated further.

Separately, a reader raised a concern on PubPeer about data presented in Figure 5E. We investigated this matter and examined the raw images carefully. We did insert the wrong image of EEA1/FAK in the vector group unintentionally. We have provided three independent replicates to the editors to explain the misuse of the image. Although the mistake does not affect the variation trend between multiple replications, we deeply apologize to readers.

All authors have agreed to the retraction.

